# Influence of different grinding degrees of fly ash on properties and reaction degrees of geopolymers

**DOI:** 10.1371/journal.pone.0282927

**Published:** 2023-03-16

**Authors:** Qingwei Sun, Siyuan Zhao, Xuzhe Zhao, Yu Song, Xinyu Ban, Ni Zhang

**Affiliations:** 1 College of Civil Engineering, Liaoning Technical University, Fuxin, China; 2 College of Civil Engineering, Guangxi Key Laboratory of Mechanics and Geotechnical Engineering, Guilin University of Technology, Guilin, China; SASTRA Deemed University, INDIA

## Abstract

This study reports the preparation of geopolymers with a mechanical performance similar to that of cement at room temperature by ground fly ash mixed with a small amount of cement. The grinding time of fly ash raw materials was 0,20,40 and 60 min, respectively. The influence of the grinding degree of the fly ash on the properties and the reaction degree of the geopolymer were investigated by XRD, SEM, EDS, and mercury compression tests. The reaction degree of the fly ash geopolymer was quantified by the selective dissolution method. Increasing the grinding degree of fly ash significantly increased the compressive strength of the geopolymer and the density of the microstructure of materials also increased. Furthermore, porosity and the average pore size decreased and the proportion of small holes in the pores gradually increased. The calculation results were in coincidence with the compressive strength test and the micro-performance test of the material, thus indicating that the selective dissolution method can reflect the influence of the grinding degree on the reaction degree of the geopolymer. Furthermore, the reaction degree of the geopolymer increased as the grinding degree of the fly ash increased. However, the growth rate of the reaction degree for the geopolymer slowed down when the fly ash was ground for more than 40 min.

## 1. Introduction

With the acceleration of social development, the demand for Portland cement in the construction industry is increasing, and the production of cement poses a great burden on the protection of the environment [1–4]. Researchers are driven to find new green materials that can partially or completely replace traditional cement. Geopolymers are new type of green cementitious material. The production process of geopolymers has lower carbon emissions than that of cement. Therefore, the development prospect of geopolymers is eminently broad [5]. Geopolymerization is a process in which aluminosilicate materials react with activators with strong bases (such as sodium silicate solution, NaOH and their combinations) to form a three-dimensional polymerization chain [6]. Compared to cement materials, geopolymers exhibit superior properties including better durability, lower shrinkage, higher strength and superior heat resistance [7–9]. Coal remains the main fuel for thermal power generation so far, and with 46% of the world ’s electricity expected to be supplied by coal-fired power by 2030 [10]. Fly ash is the main solid waste after coal combustion, which is easy to obtain and inexpensive [11,12]. Fly ash is rich in active substances such as SiO_2_ and Al_2_O_3_, which is an ideal material for preparing geopolymers.

There are three ways to activate fly ash in the process of preparing fly ash geopolymers: thermal activation, chemical activation and mechanical activation. Some studies have shown that geopolymers with similar mechanical properties as cement can be prepared by appropriately increasing the curing temperature [13,14]. On the one hand, increasing the temperature accelerates the frequency of activity between particles and improves the geological polymerization reaction speed of fly ash; on the other hand, it accelerates the evaporation of excess free water in the reaction system and promotes the hardening and condensation of geopolymers [15,16]. Thus, high-temperature curing limits the practical application of fly ash geopolymers in field construction. Therefore, in recent years, many scholars are absorbed in the preparation of fly ash geopolymers under room temperature conditions [17–19]. Compared with cement slag and other materials, fly ash has a lower activity at room temperature, so the strength of geopolymers manufactured by fly ash is generally low. Although many scholars have adopted methods for enhancing the concentration of activators or using compound activators, the actual effect is not ideal [20–22]. At present, scholars adopt the method of mixing ground slag and fly ash to improve the mechanical performances of fly ash geopolymers [23,24]. The mechanical properties of geopolymer are improved by mixing fine bottom ash-fly and fly ash [6]. Saha et al. [25] verified the influence of adding different amounts of ground slag in a fly ash geopolymer at room temperature, and the results presented that when 50% ground slag was added, the mechanical performances of fly ash were good, and the compressive strength was 78.2 MPa at 56 d. Although the incorporation of ground slag could effectively improve the geopolymeric properties, the activity of fly ash itself was not effectively stimulated and utilized at this time. The grinding of the fly ash to improve its activity is an effective way to prepare fly ash geopolymers with excellent mechanical properties at room temperature [26–28].

Although the chemical composition of the fly ash does not change after grinding, mechanical grinding destroys the dense glassy shell of fly ash particles, releases the active components inside, reduces the average particle size of fly ash particles, increases the contact area between the fly ash and activator, and increases the dissolution rate. In addition, according to the law of conservation of energy, part of the mechanical energy is transformed into kinetic energy between the particles in fly ash, which improves the activity of fly ash [29–31]. Szabo et al. [32] researched the effect of fly ash fineness on the rheological properties and microscopic morphologies of foam geopolymer paste, and the results showed that the rheological performence of the geopolymer paste changed from Bingham plastic to Newtonian liquid when ground fly ash was added; furthermore, the compressive strength of the prepared geopolymer the increased as the fine of the fly ash increased. Somna et al. [33] manufactured a fly ash geopolymer with a 28d compressive strength of 20 MPa at room temperature using 100% ground fly ash and studied the microstructure of the geopolymer by means of X-ray diffractometry (XRD), scanning electron microscopy (SEM) and energy-dispersive spectroscopy (EDS). Mechanical activation is currently the most promising method alternative to thermal activation, which reducing energy consumption by 50% -90% during processing [34]. Moreover, mechanical activation provides more possibilities for engineering sites and reduces final costs [35]. However, research on the preparation of geopolymers from grinding fly ash is not deep enough at present, especially studies on the effect of the different grinding degrees of fly ash on the related properties and reaction degrees of geopolymers. The existing research lacks a quantitative calculation method for the reaction degree of geopolymer after mechanical activation of fly ash.

This research adopts the method of mechanical activation to prepare fly ash with different grinding degrees, and fly ash geopolymer with comparable mechanical properties to cement was prepared at room temperature. The effects of grinding degree of fly ash on the fluidity, compressive strength, microstructure, crystal phase product, pore distribution and reaction degree of geopolymer were investigated from macro and micro perspectives. A quantitative calculation formula for the reaction degree of geopolymer after fly ash grinding is proposed. An objective and effective method for evaluating the effect of different grinding degrees of fly ash on the performance and reaction degree of geopolymer is provided under normal temperature conditions.

## 2. Materials and methods

### 2.1. Materials

In this passage, F-grade low-calcium fly ash produced by Fuxin Thermal Power Plant was utilized as the raw material for manufacturing geopolymers. To improve the early strength of the geopolymers, a small amount of ordinary 42.5 grade Portland cement (15%) was added. Cement was produced from Fuxin Daying Cement Plant. The basic chemical compositions of the fly ash and cement are presented in Table 1. The activator was a mixture of sodium silicate and NaOH, of which the purity of the commercial NaOH was 96% (analytically pure). A composite activator composed of sodium silicate solution and NaOH was used in this study, where the purity of NaOH produced from Liaoning Ruiquan Reagent Co., Ltd. was 96% (analytical purity). The sodium silicate solution produced from Jiashan County Yourui Refractory Co., Ltd. was transparent liquid, with a modulus of 3.3, a Baume degree of 38.5 Be, moisture content of 61.5%, a SiO_2_ mass fraction of 27.3%, and a Na_2_O mass fraction of 8.54%.

**Table 1:** Chemical composition of the fly ash and cement.

Content (100%)	SiO_2_	Al_2_O_3_	CaO	Fe_2_O_3_	MgO	Na_2_O	K_2_O	SO_3_	Loss
Fly ash	52.31	28.59	4.01	6.22	2.05	/	1.62	1.79	3.41
Cement	23.22	4.51	60.47	2.33	1.16	0.87	1.23	4.22	1.99

**Fig 1:**
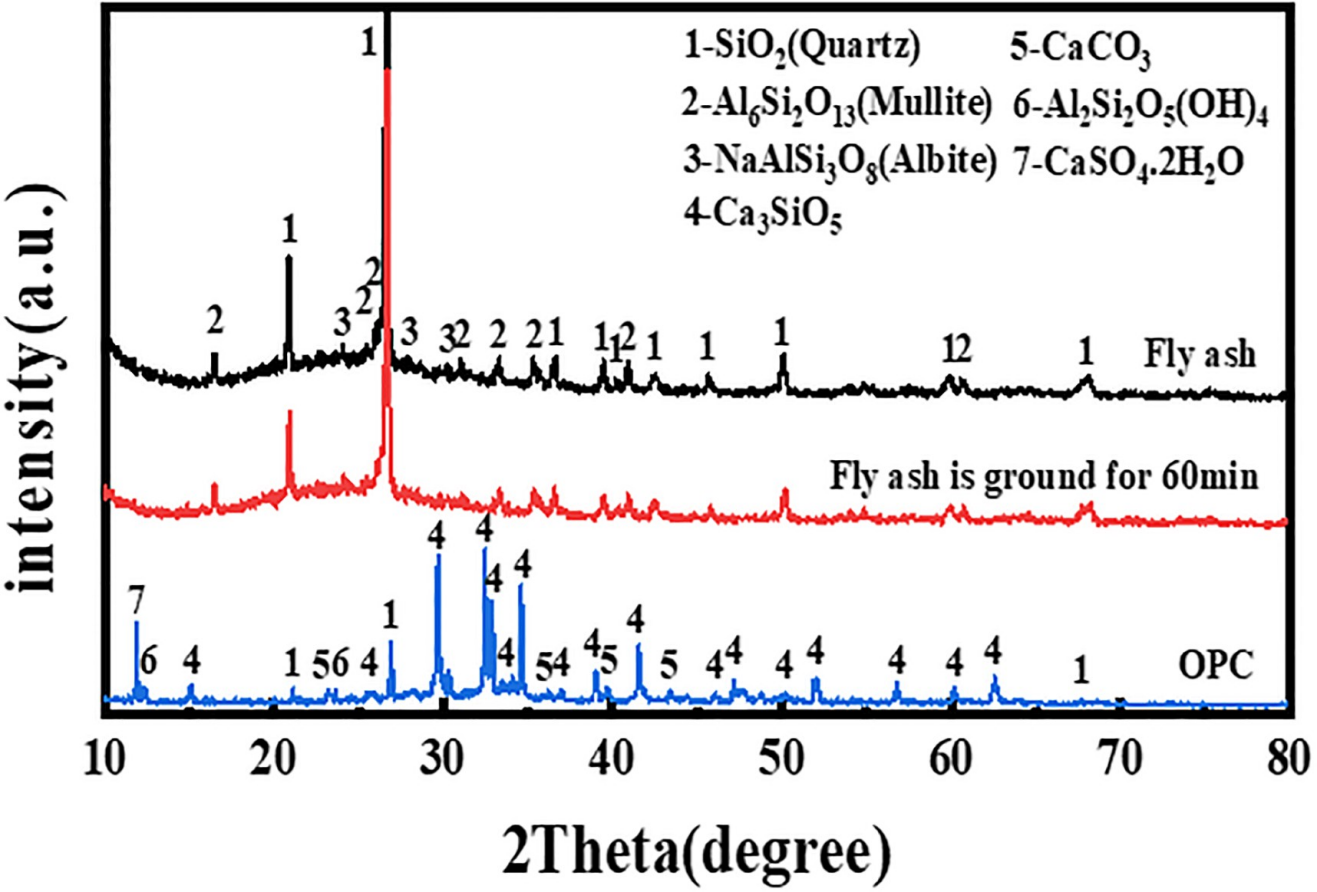
XRD patterns of the raw materials.

The XRD patterns of the fly ash and cement are depicted in Fig 1. The results of the phase analysis show that the main crystalline phases of the fly ash were quartz, mullite and albite. After mechanical activation, the diffraction peak intensities of the crystalline phases did not change markedly, indicating that the crystalline phase types in the fly ash did not change.

### 2.2. Sample preparation

Mechanical activation process is that the raw materials of fly ash are mechanically activated by LSYM-5L basket grinder produced in Nanjing, China. The raw materials of fly ash are ground by dispersed blades, and the grinding speed was stable at 2000r / min. Based on the previous test, it was found that the short grinding time interval had no obvious effect on the performance of the sample. Fly ash raw materials undergoes three different activation grinding times: 20, 40 and 60 min to prepare for benchmark tests. The particle size of fly ash can be reduced by mechanical activation, and the dense glass phase shell on the surface of the particles can be destroyed. The pozzolanic activity of fly ash increases significantly.

In this study, four groups of geopolymer ratios were designed, in which fly ash and cement were composed at a fixed ratio of 85:15, and the water-binder ratio was fixed at 0.4. Another group of cement samples was utilized as a reference control, and the detailed ratio of the five samples is shown in Table 2. F0, F1, F2 and F3 represent the grinding time of fly ash at 0 min, 20 min, 40 min and 60 min, providing 4 different particle sizes of fly ash. The optimal concentration of OH within the activator is 14.2 mol/L based on a preliminary test. SiO_2_/Na_2_ and H_2_O/Na_2_O is 1.8 and 3.9, respectively. FG0, FG1, FG2 and FG3 represent the preparation of fly ash based geopolymers with original fly ash and fly ash with different grinding time as raw materials after adding 15% cement [36].

**Table 2:** Experimental design scheme.

Sample	Cement(g)	Fly ash(g)		Grinding time(min)	NaoH(g)	Water glass(g)	Water-binder ratio
		Raw(g)	Ground(g)				
OPC	340	/	/	/	/	/	0.4
FG0	50	290	0	0	29.5	212	0.4
FG1	50	0	290	20	29.5	212	0.4
FG2	50	0	290	40	29.5	212	0.4
FG3	50	0	290	60	29.5	212	0.4

The sample preparation process: (1) The preparation of the activator needs to be completed 24 hours in advance. According to the ratio requirements, NaOH, water glass and added water are weighed respectively, mixed and stirred until transparent, sealed and stored; (2) Weighing fly ash and cement as shown in Table 2, pouring them into the NJ-160A slurry mixer and stirring them slowly for 30 s to mix the two materials evenly. (3) The prepared activator solution was poured into the slurry mixer. The slurry was slowly stirred for 2 min, and the mixer was closed for 30 s. During the static process, the slurry attached to the fan blade and the pot wall was scraped into the bottom of the pot, and then quickly stirred for 2 min to prepare the geopolymer. (4) The fluidity test was carried out first. And then the slurry was loaded into a 40 mm × 40 mm × 40 mm cube mold. Each group was made of 6 test blocks, covered with plastic wrap and placed indoors. After 24 hours, the mold was removed and placed in a curing box with a temperature of 20°C and a humidity of 98%.

The experimental part of this study was completed in the civil engineering laboratory of Liaoning Technical University in Fuxin City, Liaoning Province.

### 2.3. Testing

#### 2.3.1. Measurement of particle size

A MasterSizer 3000 laser particle size analyser produced in the UK was utilized to analyze and test the particle size of the fly ash with different grinding degrees. The dry method was utilized to test the particle size. A sample weight between 2~3 g was selected. This method mainly uses a laser beam to measure the particles size of fly ash. The laser beam is scattered to different angles when meeting differently sized fly ash particles. Low-angle scattered light is caused by large particles, while high-angle scattered light is caused by small particles. The different intensities of the scattered light represent the number of fly ash particles with different sizes.

#### 2.3.2. Liquidity

Referring to GB/T 8077–2012 "Test Method for Homogeneity of Concrete Admixtures", the materials were mixed according to the defined ratio. The mixture was then placed into an NJ-160A pure slurry mixer (produced Wuxi, China), stirred, and poured immediately onto a truncated cone mould with a height of 60 mm, an upper diameter of 36 mm and a lower diameter of 60 mm. The truncated cone mould was placed on a 400 mm × 400 mm × 5 mm glass plate and then lifted vertically. The slurry was allowed to flow onto the glass plate for 30 s, and then a ruler was immediately utilized to measure the dimensions in the perpendicular directions. The diameter of the sample in the perpendicular direction was averaged to calculate the fluidity of the pure slurry.

#### 2.3.3. Compressive strength

The compressive strength of different geopolymer samples at different ages was tested by TAW-2000 instrument from Changchun, China. Reference was made to GB / T50081-2019 "Standard for Test Methods of Physical and Mechanical Properties of Concrete". Compressive strength tests were carried out on 40 x 40 x 40 mm cubic specimens. After specimen testing, the average of the two (6 samples) groups of data was obtained to reduce experimental error.

#### 2.3.4. X-ray diffractometry (XRD), scanning electron microscopy (SEM) and energy-dispersive spectroscopy (EDS)

The phase analysis of the fly ash, cement and geopolymer was carried out with a Bruker D8 Advance X-ray diffractometer produced in Germany at a scanning speed of 5°/min and a scanning angle of 10–80°. A Zeiss Gemini 300 scanning electron microscope produced in Germany was utilized to observe the micro-morphology of fly ash with different grinding degrees, and EDS of the prepared geopolymers was used to analyse the energy spectrum of the geopolymer and observe its elemental distribution.

#### 2.3.5. Mercury intrusion method

The pore size and distribution of the geopolymer prepared from four fly ash samples with different grinding degrees were detected by an Autopore IV 9510 mercury injection apparatus produced the United States. This method applies pressure to mercury promoting its diffusion; the resistance to enter pores can be used to measure the pore aperture and the pore distribution. The fractal dimension of the pore size distribution can be further calculated from the mercury injection test data.

#### 2.3.6. Selective dissolution method

Cement and its products along with aluminium silicate polymer can be completely dissolved in hydrochloric acid. In unreacted fly ash, only trace alkaline minerals are dissolved in hydrochloric acid, and the reaction amount is negligible; therefore, unreacted fly ash is considered to be insoluble in hydrochloric acid, allowing the selective dissolution method was utilized to measure the content of unreacted fly ash [37–40]. The complete test block is removed and ground after crushed, which is weighed 10 g as a test sample. The samples were dissolved in 1:20 HCl (vol%). Briefly, 1 g of each sample was dissolved in 250 ml of the hydrochloric acid solution. After being oscillated (SHZ-82 gas bath thermostatic oscillator from Changzhou, China) for 3 h, the samples were centrifuged (LD-5 table centrifuge from Changzhou, China) for 10 min. Then, the solution was filtered with slow filter paper, leaving unreacted fly ash particles, which were cleaned with deionized water until the pH of the solution after cleaning was neutral. The above steps were repeated three times, and then the unreacted fly ash particles were placed in a drying box until the weight no longer changed. Record the weight at this time and calculate.

## 3. Results

### 3.1. Mean particle size of fly ash

**Fig 2:**
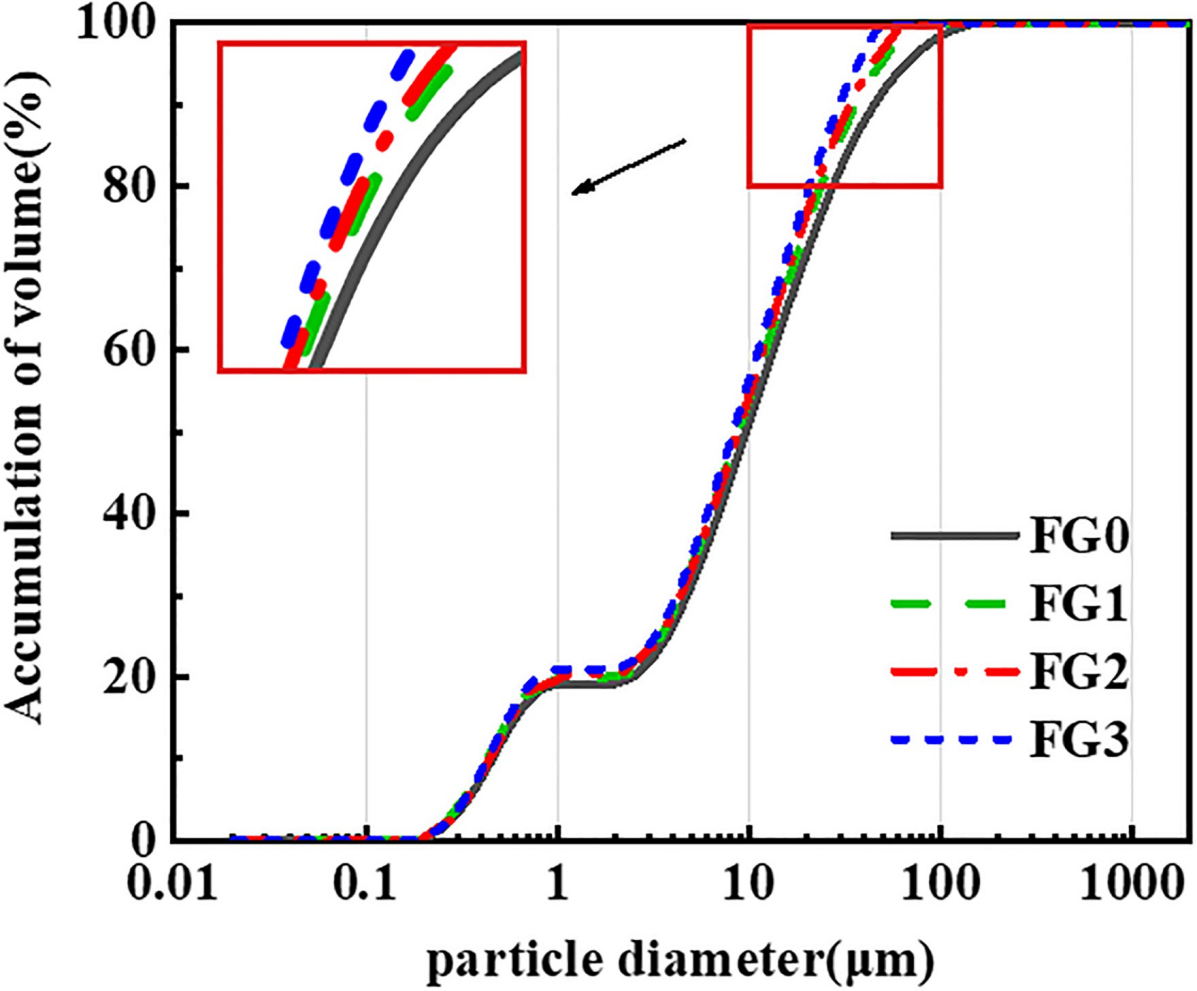
Particle size distribution diagram.

The raw material of fly ash was preprocessed by an LSYM-5L basket-type wet mill grinding machine, and fly ash samples with different grinding degrees were prepared by controlling the grinding time. The analysis results of the particle size for four fly ash samples with different grinding degrees are presented in Fig 2. The trends of the particle size distribution of the four fly ash samples were similar, with main distributions of 0.1–1 μm and 2.5–145 μm. The four kinds of fly ash rendered to 1–2.5 μm showed small changes for the content of fly ash particles in this particle size range.

The particle size distribution range inside fly ash was large, and after mechanical grinding, the number of large particles in fly ash was greatly reduced, while the number of small particles in fly ash showed little change. The formula for calculating the average particle size of fly ash is as follows:

$$Q=\bar{\sum_{i=0}^{100}D(\text{i})}$$ (1)

The particle size classification data of the four fly ash samples are presented in Table 3. The average particle size of the fly ash was 25.7845 μm before grinding. With an increasing milling time, the average particle size of fly ash was 10.5213 μm after 60 min.

**Table 3:** Scale of the characteristic size.

Sample	DX (10) (μm)	DX (50) (μm)	DX (90) (μm)
F0	0.449	9.34	41.7
F1	0.467	8.95	31.4
F2	0.47	8.06	26.5
F3	0.438	7.33	22.8

**Fig 3:**
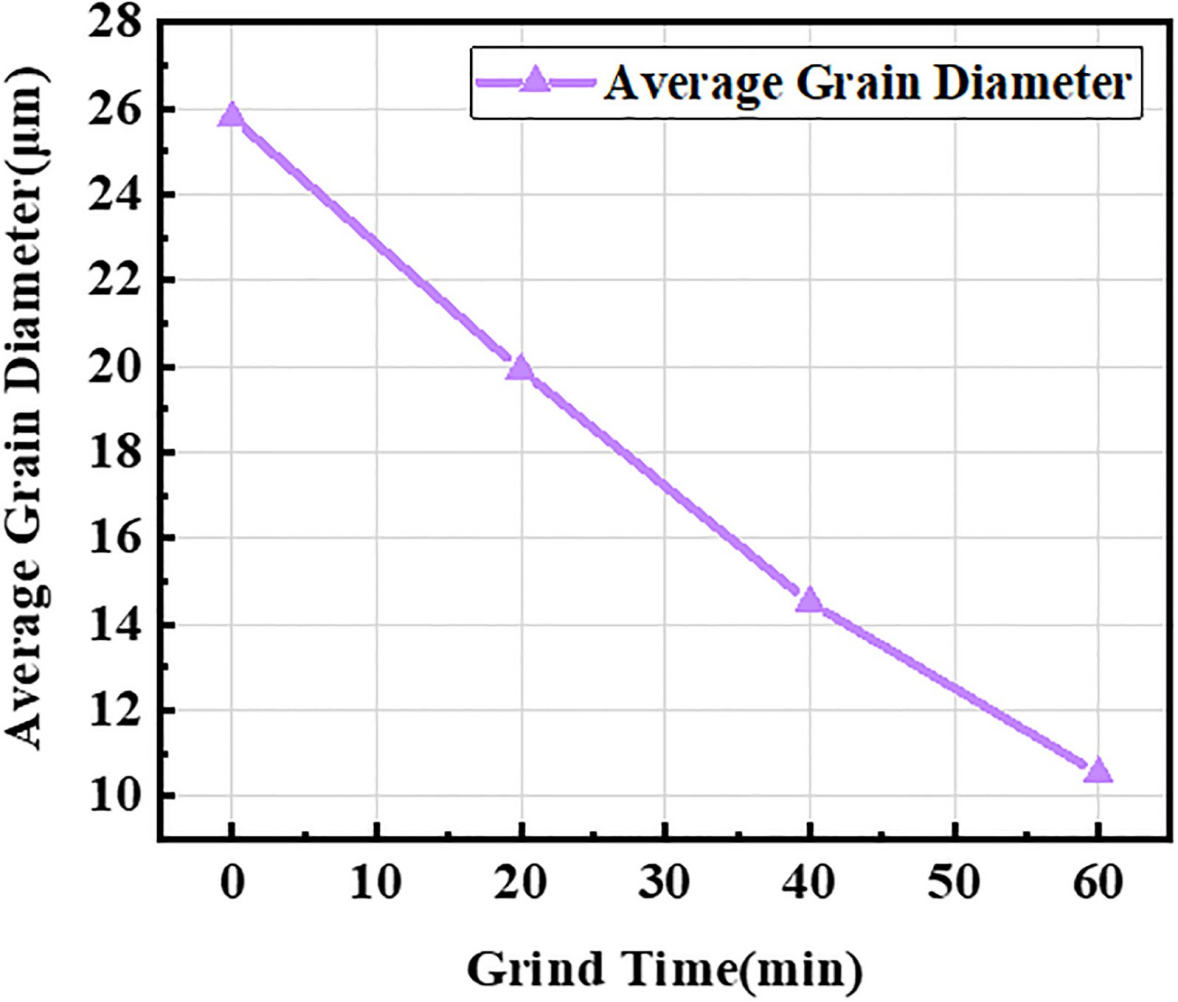
Relationship between grinding time and average particle size of fly ash.

The curve showing the relationship between the average fly ash particle size and grinding time is shown in Fig 3. The average particle size of fly ash gradually decreased as grinding time increased from the test results. However, the curve was not a strictly linear function. As the grinding time increased, the average particle size of the fly ash gradually decreased.

### 3.2. Slurry fluidity

**Fig 4:**
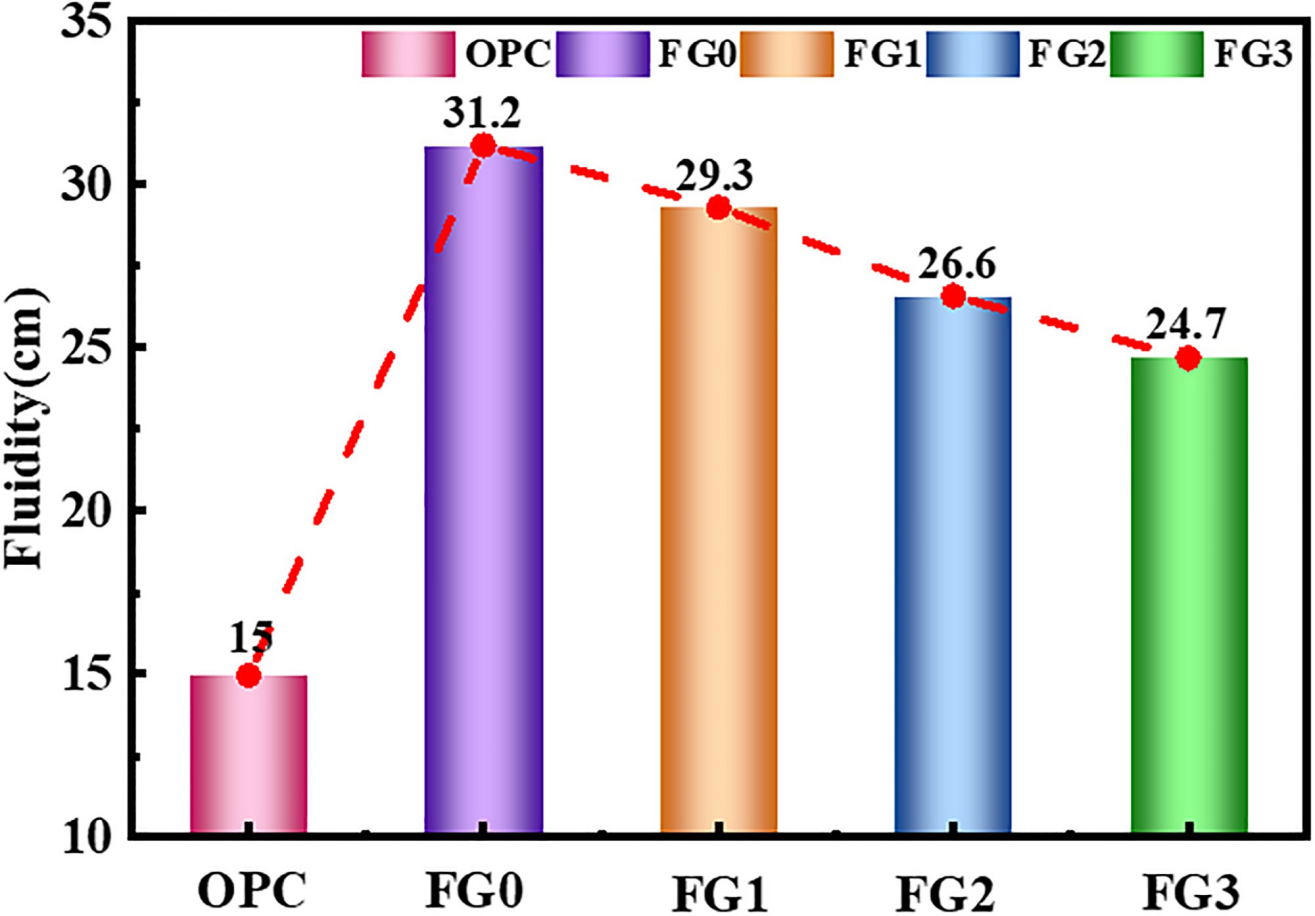
Net slurry fluidity.

As shown in Fig 4, the fluidity of FG0 was 31.2 cm, which was much higher than the fluidity of ordinary Portland cement (OPC, 15 cm). This result is due to the microscopic spherical particle morphology of fly ash, which provides fly ash with good fluidity and has been confirmed in many previous studies and practical field applications. The fluidity of the prepared fly ash based geopolymer decreased as the grinding degree of the fly ash increased. In particular, the fluidity of FG3 decreased the most (24.7cm), which was 20.8% lower than that of FG0 since the fineness of fly ash increased after grinding. Increasing the total surface area leads to the increase of water demand, thus resulting in a decrease in sample fluidity.

Furthermore, some of the smooth surface fly ash particles were broken into irregularly shaped fine particles during the grinding process. As the grinding time increased, the smooth fly ash particles decreased and the small particles with irregular shape gradually increased, which increased the internal friction and resistance during slurry flow. Therefore, the macro performance is reduced fluidity. However, FG3 still had good fluidity which was 64.7% higher than that of OPC.

### 3.3. Compressive strength

**Fig 5:**
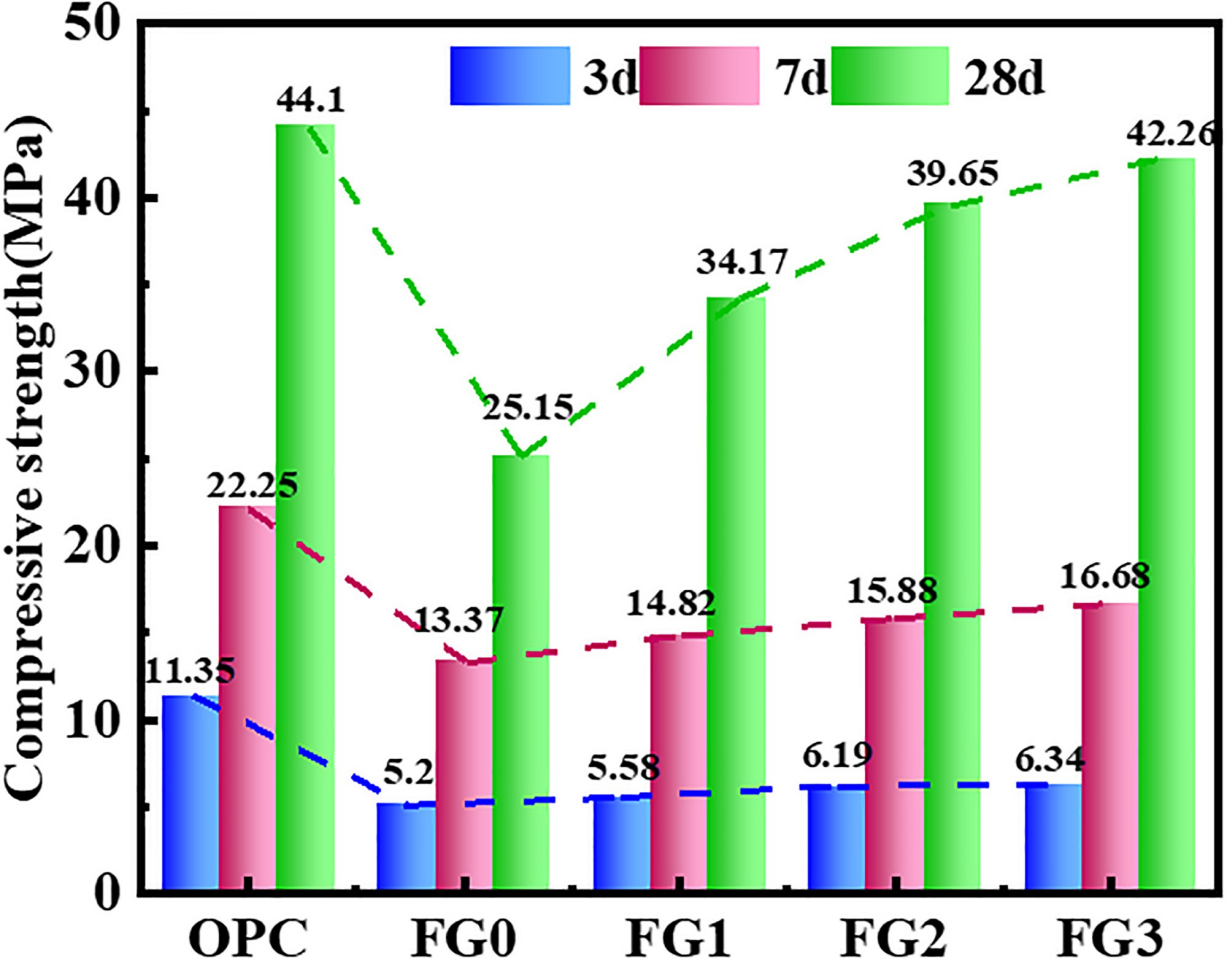
Compressive strength of cement slurry and geopolymers.

The reaction mechanism of the geopolymer itself was relatively complex, the change in various factors had a large influence on the reaction process of the whole system, and the compressive strength was the most important index for reflecting the properties of the geopolymer. The compressive strength results of the cement and fly ash based geopolymer in each group are depicted in Fig 5. The test results showed that compared with OPC, when both the raw fly ash and ground fly ash was used to prepare fly ash based geopolymers, they demonstrated low early strengths. For instance, the 3-d compressive strength of OPC was 11.35 MPa, while the compressive strengths of the FG0, FG1, FG2 and FG3 at a same age were 45.8%, 49.16%, 54.5% and 55.8% of the OPC value, respectively. There was little difference in the compressive strength of the four fly ash based geopolymers at the initial reaction stage. Although the fineness of the fly ash was improved through mechanical activation for FG1-FG3, the reaction degree of fly ash based geopolymers at the initial reaction stage was still low.

As the reaction proceeded, the mechanical activation effect of fly ash began to gradually appear. At 7 d, the compressive strengths of FG0, FG1, FG2 and FG3 were 60%, 66.6%, 71.4% and 75% of the OPC compressive strength, respectively. At 28 d, the compressive strengths of FG0, FG1, FG2 and FG3 were 57%, 77.48%, 89.9% and 95.8% of the OPC compressive strength, respectively, and FG3 was close to the strength level of OPC under the same preparation conditions. According to the data in the table, the growth rate of the compressive strength of the four fly ash based geopolymers was basically the same at the initial reaction stage, and the growth rate of the compressive strength of the fly ash based geopolymers was slightly higher than that of cement from 3 d to 7 d. From 7 d to 28 d, the growth rate of the compressive strength for the fly ash based geopolymer that prepared by raw fly ash slightly decreased, while the growth rate of the fly ash based geopolymer prepared with ground fly ash was significantly improved. Thus, as the grinding degree of fly ash increased, the growth rate of the compressive strength for the fly ash based geopolymer clearly improved [41,42].

All in all, the compressive strength of each age was on the rise with the increase of grinding degree and the compressive strength of 28 d age was more obvious. The increase in compressive strength can be attributed to two main reasons: 1) As the degree of grinding increased, the average particle size of fly ash particles decreased significantly. Smaller particles gradually filled the pores between larger particles. The filling of internal pores were gradually improved, and the internal structure of the fly ash base polymer became more dense as the reaction process proceeded. 2) With the gradual destruction of the glass phase shell on the surface of the fly ash particles, the fly ash particles were broken into a large number of small particles. At this time, the specific surface area of the particles increased, which increased the contact area between the particles and the alkali solution. The erosion ability of the alkali solution to the fly ash particles increased, which increased the reaction rate of the geological polymerization and increased the reaction degree of the fly ash-based geopolymer.

### 3.4. Microscopic performance analysis

#### 3.4.1. XRD

**Fig 6:**
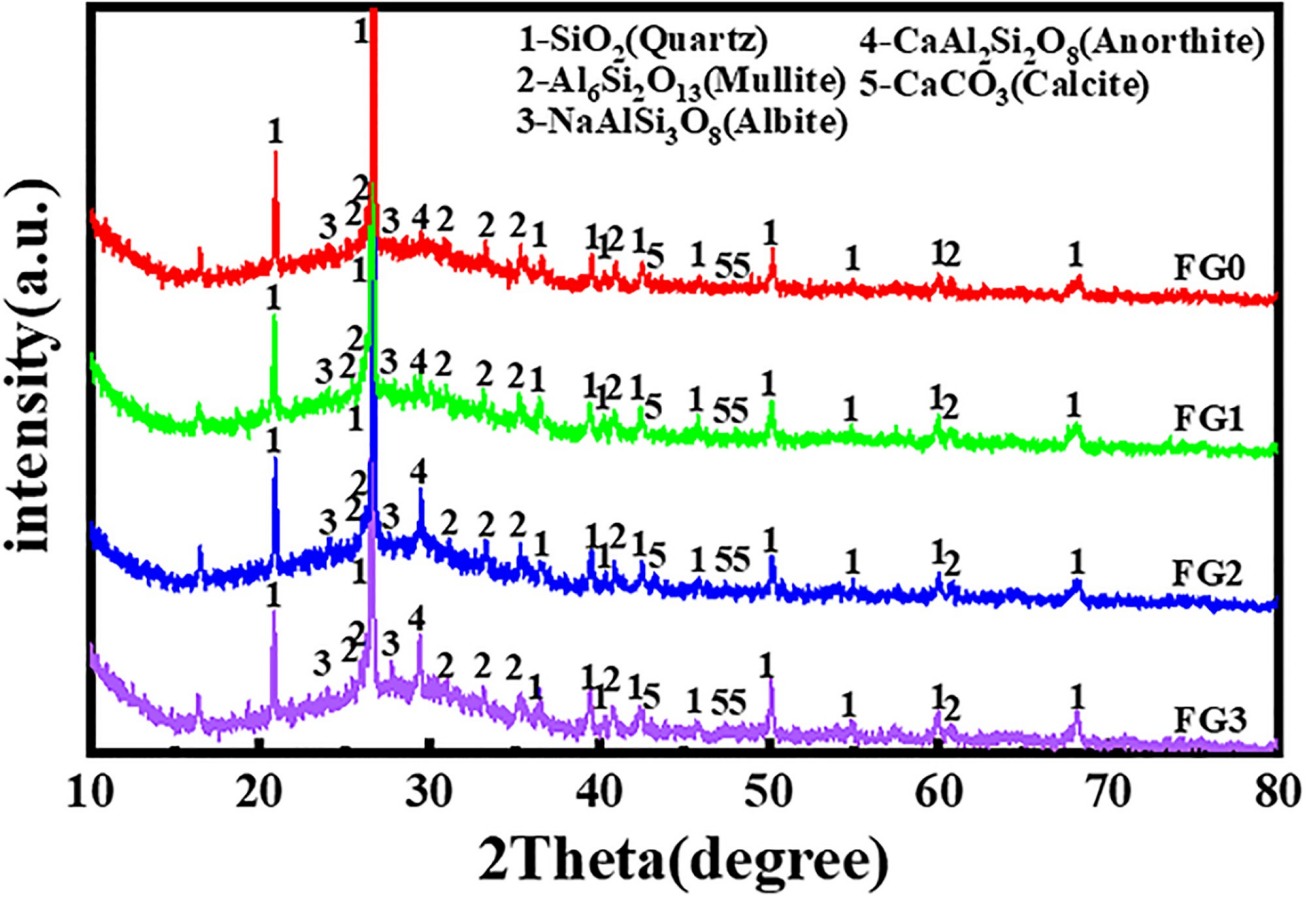
XRD of geopolymer at 28 d.

XRD images of the four fly ash based geopolymers are presented in Fig 6. The quartz phase in fly ash dissolved slowly in a highly alkali environment; thus, the diffraction peak value of quartz in the geopolymer was lower than that of quartz in the raw fly ash. In addition, due to a slight amount of cement was added to the raw material, a small amount of the calcium feldspar phase was found at 2θ = 20°, and its diffraction peak value was approximately 100–160 a.u. An extremely small amount of the calcium carbonate phase was observed at approximately 2θ = 45° and 2θ = 50°, and its diffraction peak value was approximately 50 a.u. The crystal phase diffractin peaks of mullite and albite in the geopolymer had no change compared with that of fly ash.

Generally, the XRD patterns of the four fly ash based geopolymers were basically the same. The difference was that the diffraction peak intensity of the quartz phase in the different polymer samples was different from that of the respective raw fly ash materials, and the degree of decrease in the diffraction peak intensity of the geopolymer quartz phase decreased as the grinding degree of the raw fly ash material increased. At 2θ = 26.6°, for example, the FG0 quartz phase diffraction peak intensity decreased by nearly 600 a.u. compared to that of F0, and the FG3 quartz phase diffraction peak intensity fell by nearly 300 a.u. compared to that of F0, other diffraction peak intensities of the lower quartz rule position were the same. This result may be because, in F0, all the fly ash particles were undisturbed. Thus, the activator needed to erode, dissolve and destroy the quartz-rich glassy phase shell of the fly ash particles before geopolymerization could occur with the active substances inside the fly ash, making the peak value of the FG0 quartz phase largely decrease; furthermore, the consumption of OH- in the reaction system increased, decreasing the degree of geopolymerization of the materials. In FG3, because the shell of the fly ash particles was destroyed by mechanical grinding in advance, the OH- in the activator could be directly polymerized with the active substances in fly ash, making the degree of the geopolymerization of materials more sufficient, while the shell of the fly ash was less eroded by OH-. This mechanism is why, compared with FG0, the macro-compressive strength of FG3 was higher, while the diffraction peak of the quartz phase showed less of a decrease. From the crystalline phase reaction product, the particles in the unground fly ash were not different from those in the ground fly ash and included: SiO_2_ (quartz), Al_6_Si_2_O_13_ (mullite), NaAlSi_3_O_8_ (allbite), CaAl_2_Si_2_O_8_ (anorthite), and CaCO3 (calcite).

#### 3.4.2. SEM

**Fig 7:**
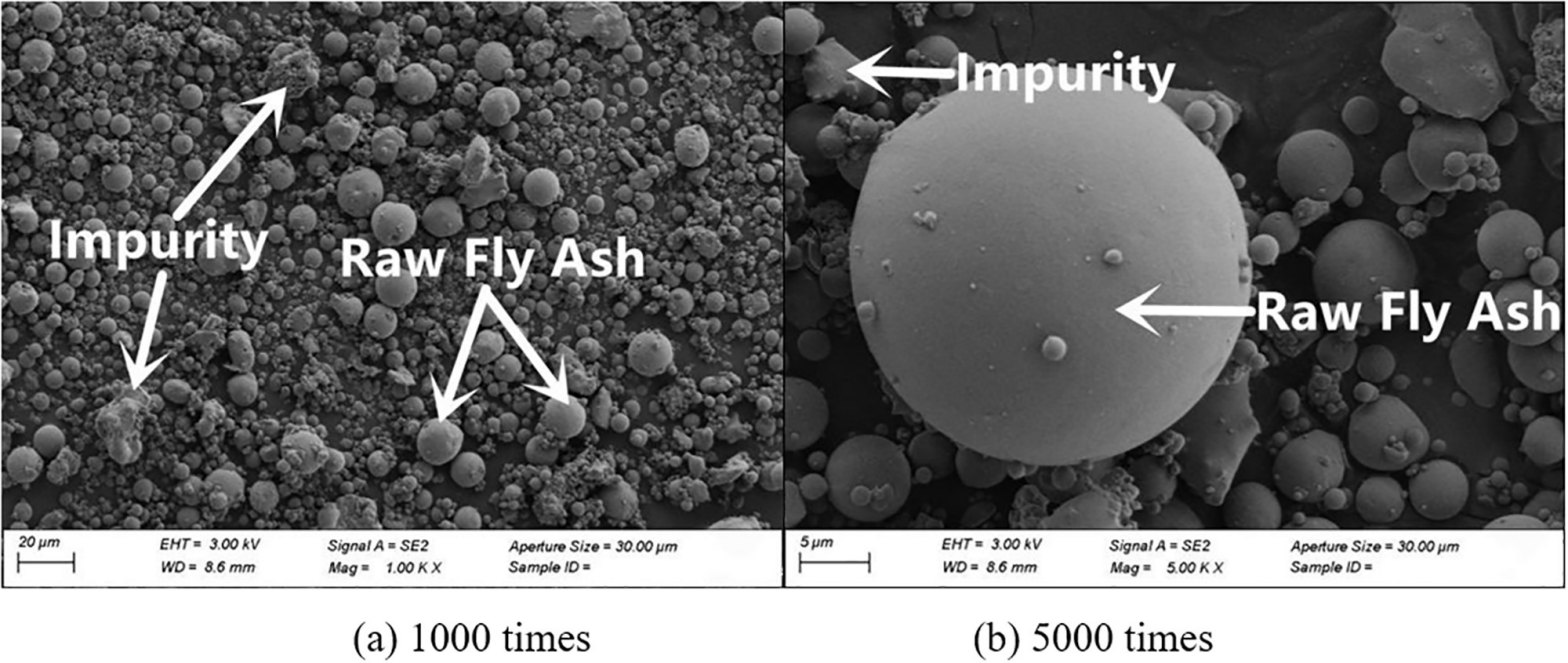
Fly ash microstructure before grinding.

**Fig 8:**
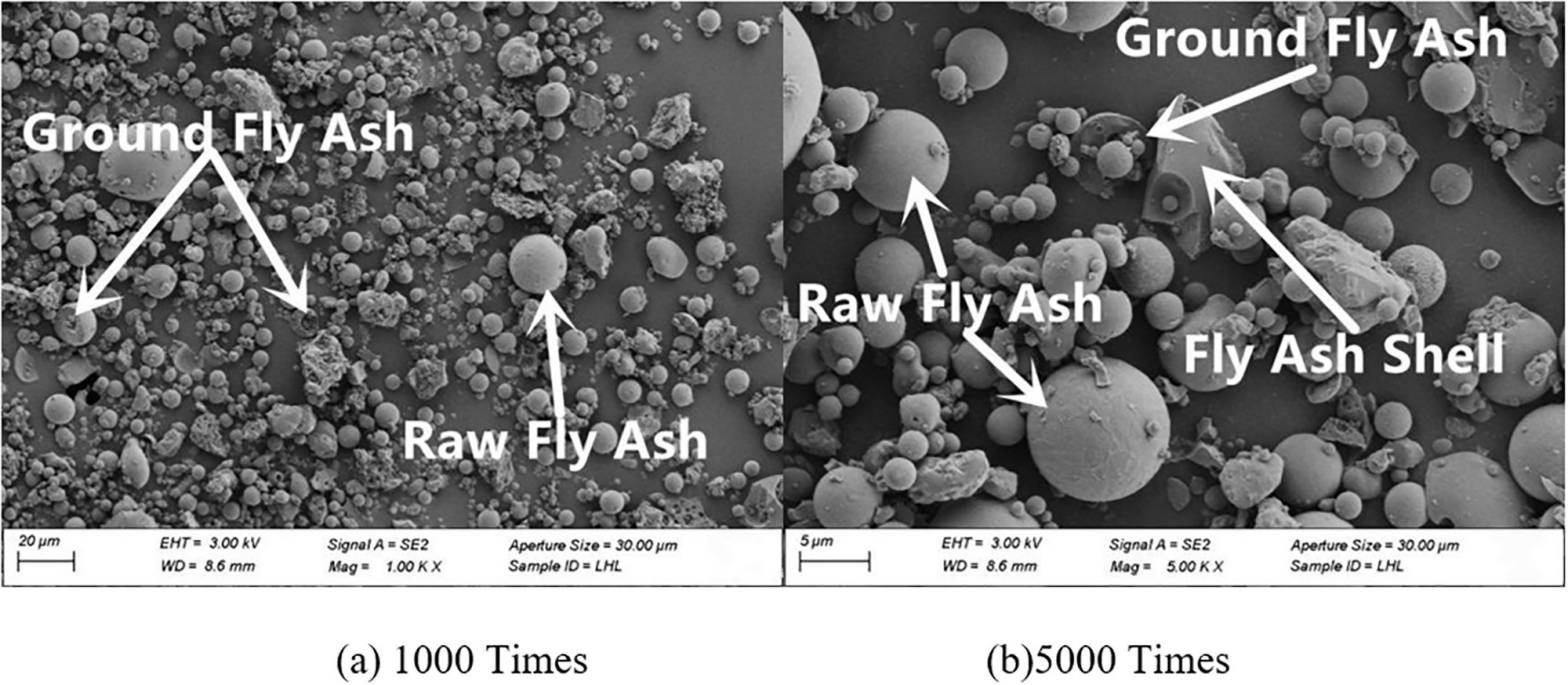
Fly ash microstructure in grinding for 20 min.

**Fig 9:**
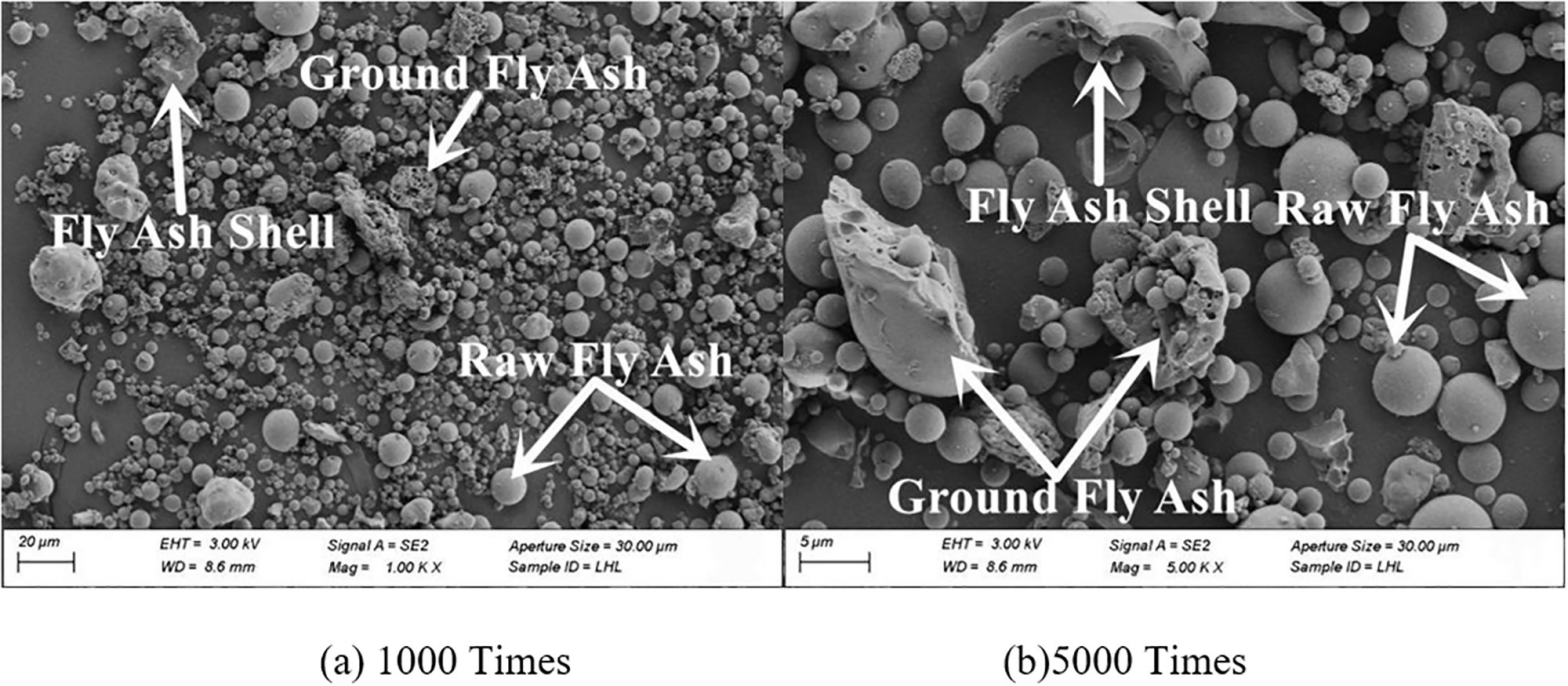
Fly ash microstructure in grinding for 40 min.

**Fig 10:**
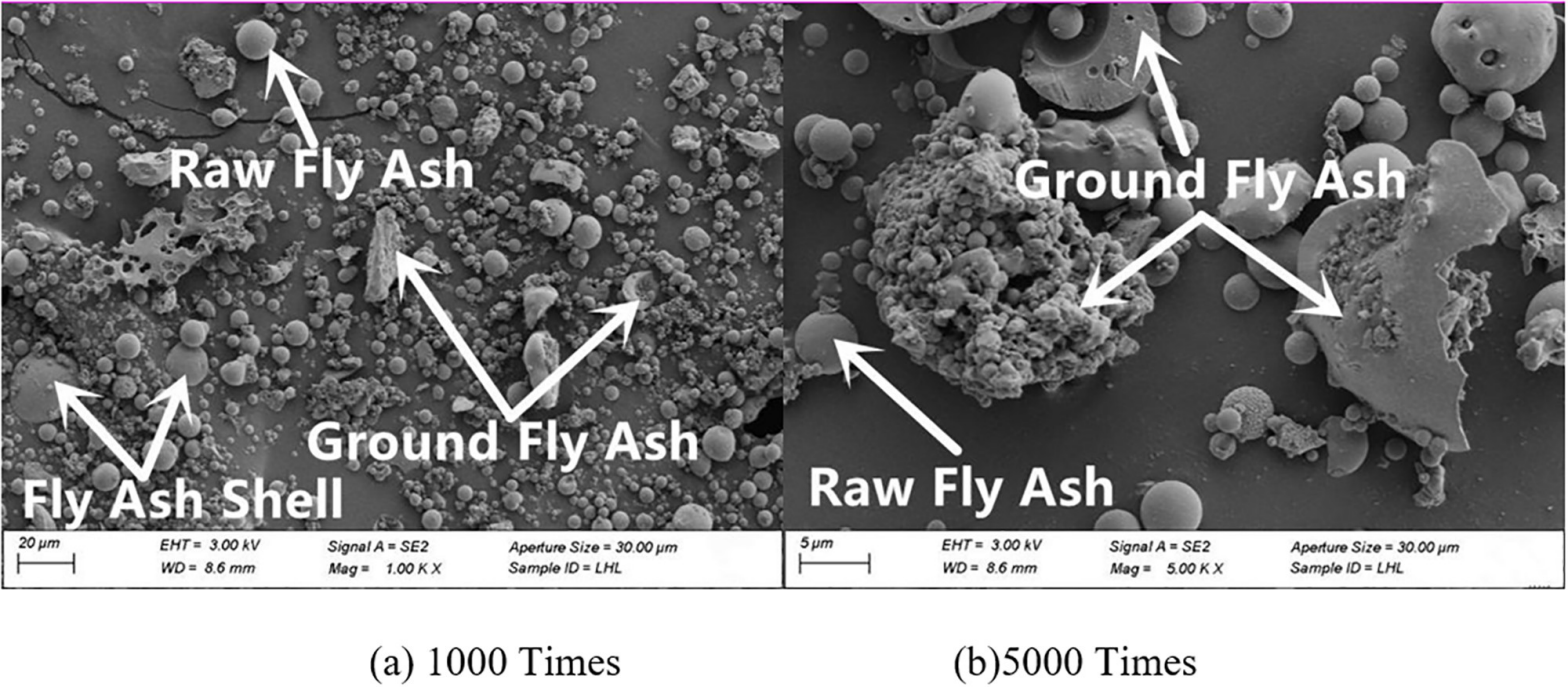
Fly ash microstructure in grinding for 60 min.

The microscopic morphology of raw fly ash consisted of many small spherical particles of different sizes, as presented in Fig 7. The microscopic morphologies of fly ash with grinding times of 20 min, 40 min and 60 min are shown in Figs 8–10. It can be clearly observed from the figures that with the increase in grinding time, the regular spherical particles in the fly ash significantly decreased, while the content of broken and irregular particles gradually increased. For instance, by comparing raw fly ash and fly ash ground for 60 min, masses of complete fly ash particles with a large particle size could be observed in the raw fly ash samples, whereas most of the fly ash particles in the sample ground for 60 min were broken with a small particle size.

**Fig 11:**
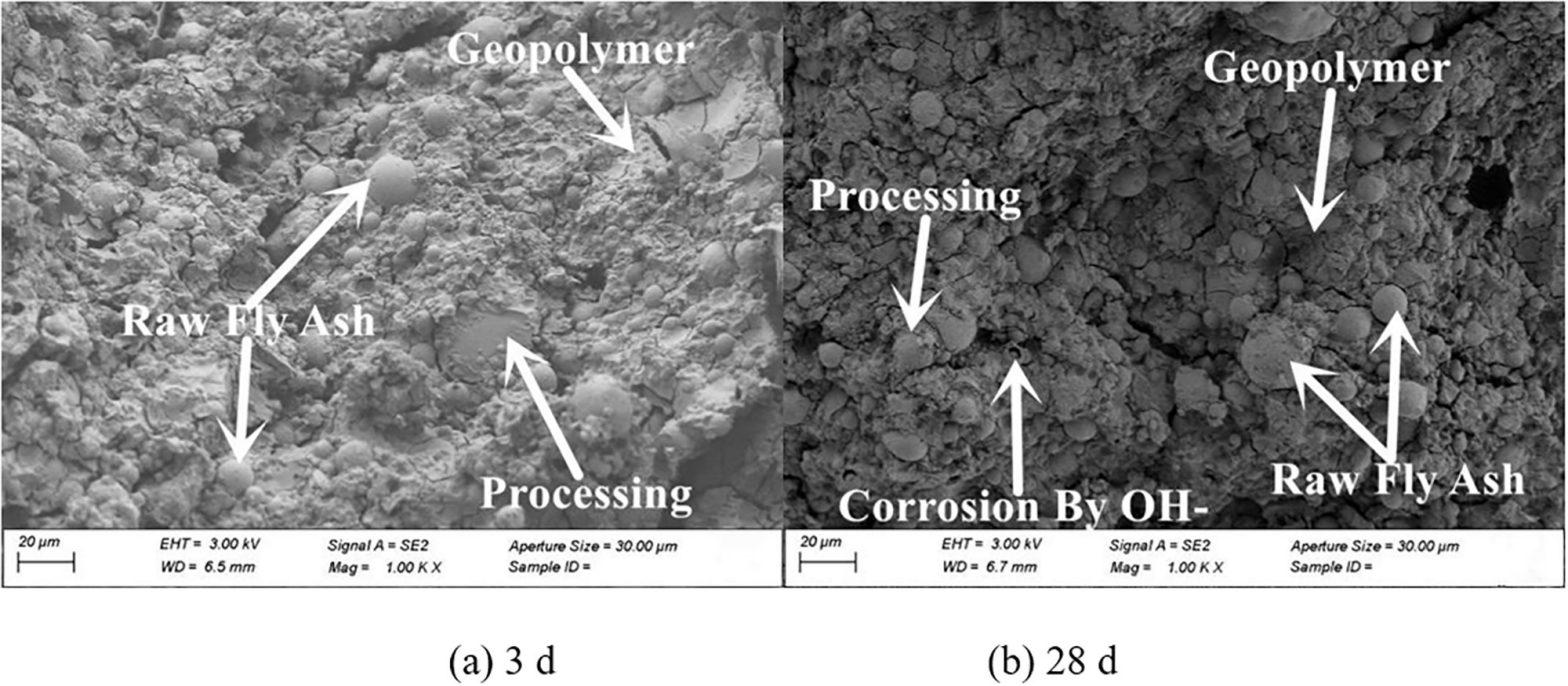
Microscopy analysis of FG0 at 28 d.

**Fig 12:**
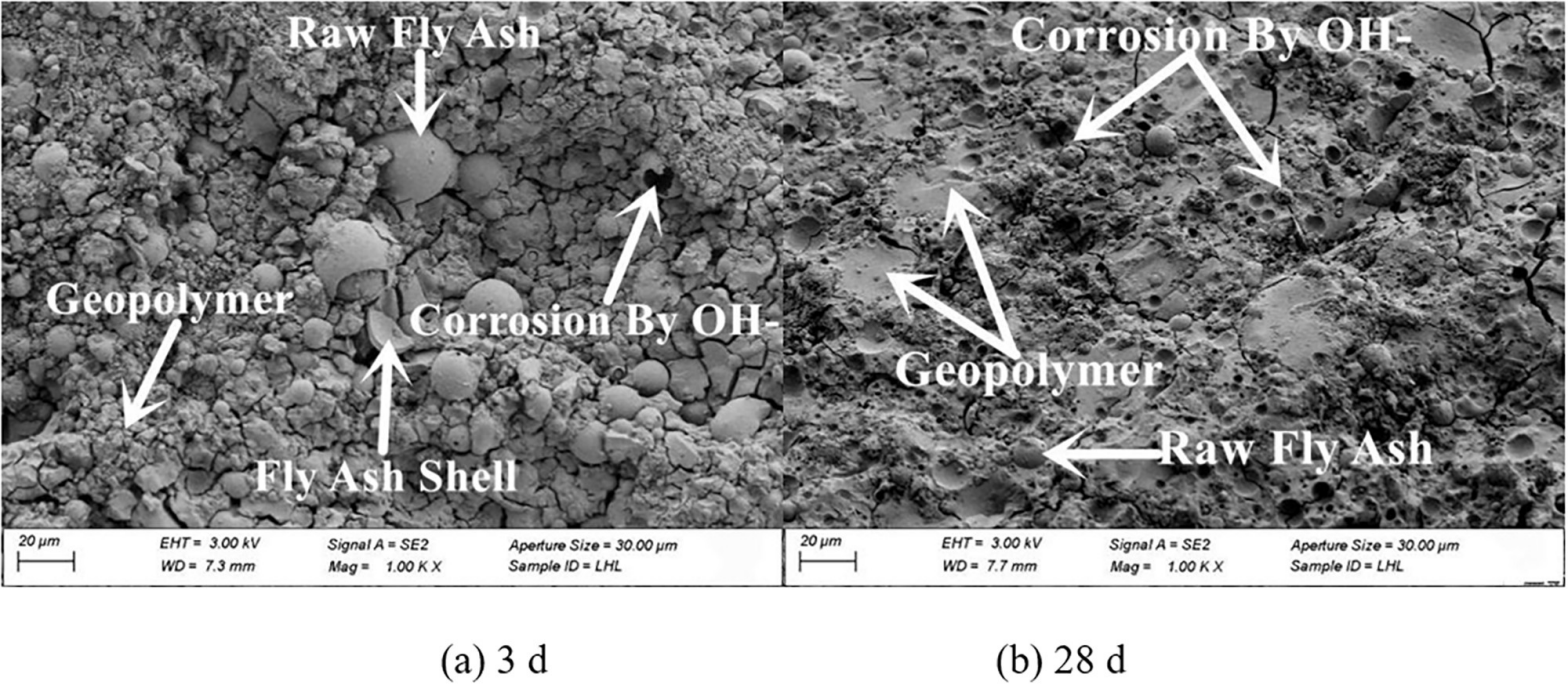
Microscopy analysis of FG1 at 28 d.

**Fig 13:**
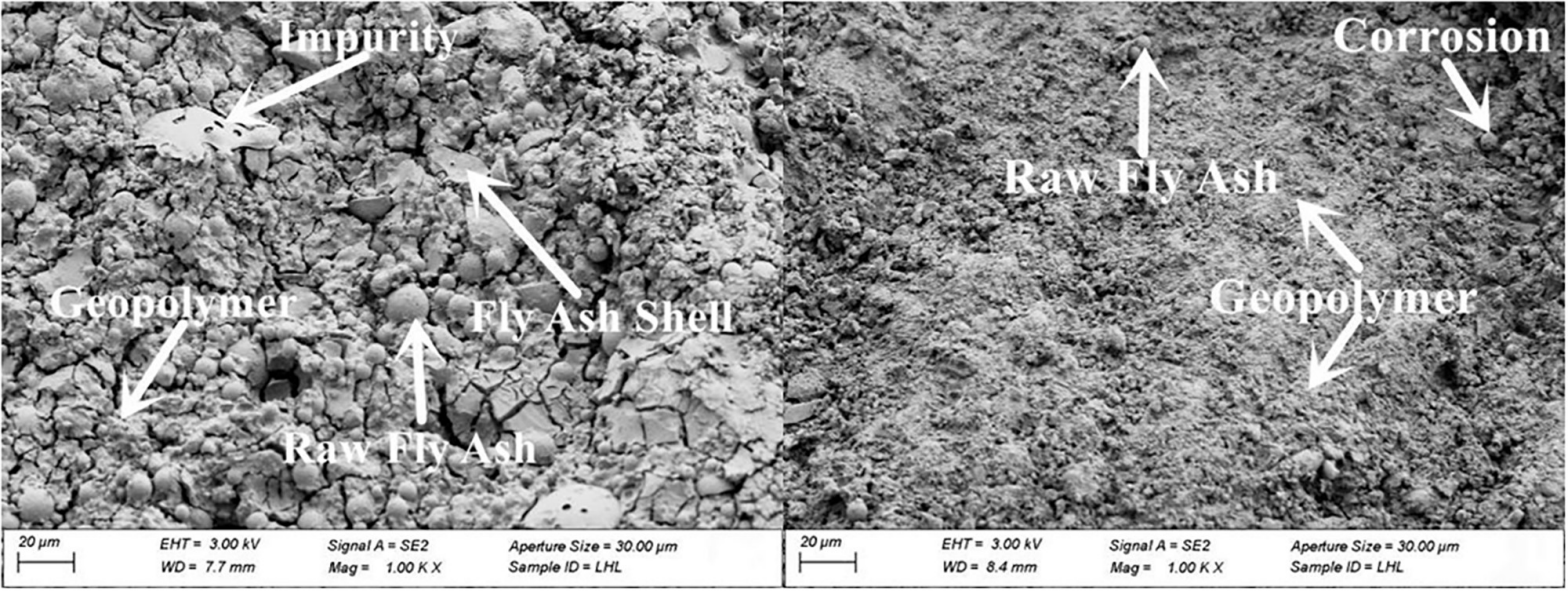
Microscopy analysis of FG2 at 28 d.

**Fig 14:**
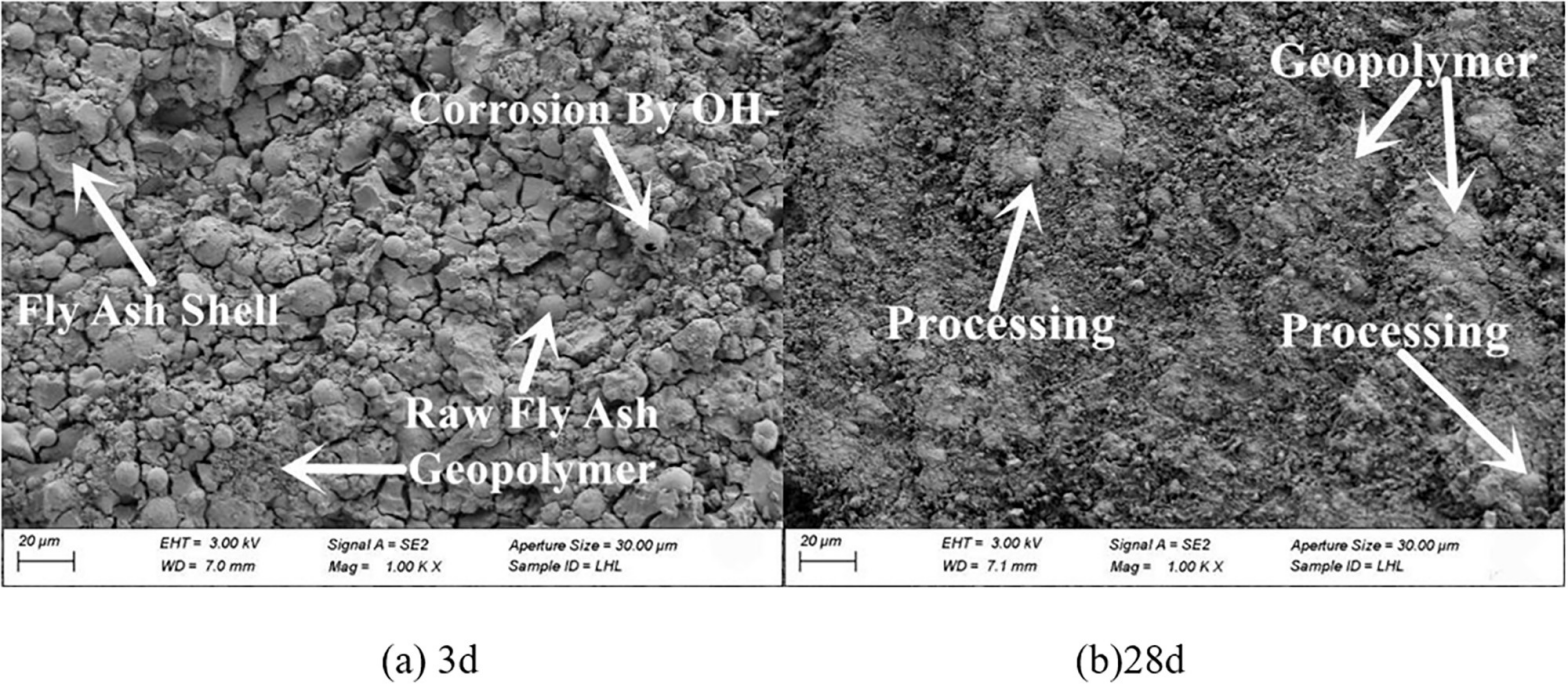
Microscopy analysis of FG3 at 28 d.

The micro-morphologies of the four fly ash based geopolymers are presented in Figs 11–14. Increasing the grinding time of fly ash resulted in a decrease in the unreacted fly ash particles and an obvious increase in the reaction products. The structure gradually changed from loose to compact. At 28 d the microstructure of the fly ash based geopolymer affected by the grinding degree of the fly ash was significantly greater than that at 3 d. At 3 d, masses of unreacted fly ash particles were observed in the geopolymer, and the quantity of unreacted fly ash particles in the sample with a large degree of grinding was reduced and the overall difference was relatively small. At 28 d, the difference in the effect of grinding degree on the product gradually became more significant. The unreacted fly ash particles significantly decreased and the reaction products of the fly ash increased as the grinding degree increased. The gel-like reaction products were generated in large quantities, the structural pores and cracks decreased, and the structure became dense.

#### 3.4.3. EDS

**Fig 15:**
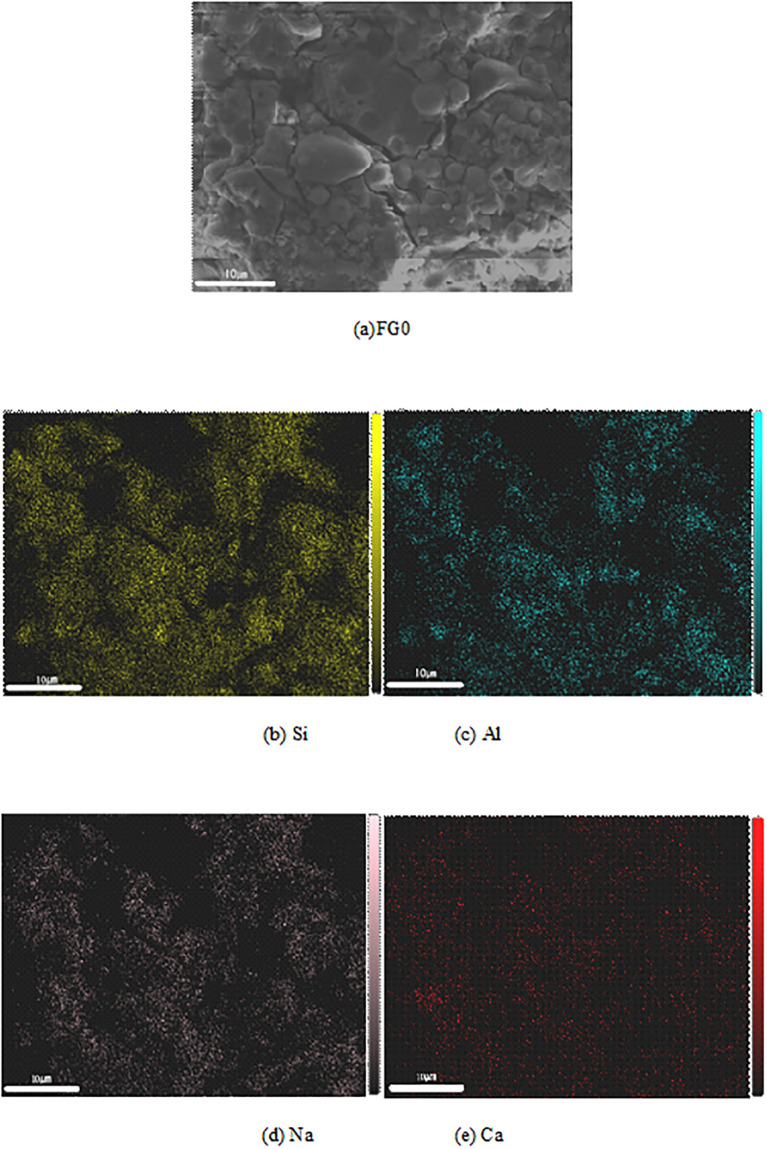
Elemental distribution of FG0.

Combined with the results of the research on the compressive strengths and microstructures of the four fly ash based geopolymers, two typical fly ash based geopolymers at the age of 28 d, FG0 and FG3, were selected as the research objects. The influence of the grinding degree on the distribution of Si, Al, Na and Ca in the fly ash based geopolymer was studied by surface scanning. The brightness of an elemental spot in the image represents the intensity of its electrical pulse signal. The higher the elemental content, the stronger the electrical pulse signal and the brighter the spot. When surface scanning FG0 and FG3, they show typical microscopic morphologies, such as unreacted fly ash particles, reacted fly ash and geopolymer gel. As shown in Fig 15, the FG0 scanning results indicate that the light spots of Al and Si are bright, widely distributed and basically coincide with each other, indicating that Al and Si mostly exist in combined forms inside the fly ash based geopolymer. The distribution of Na partially overlaps with that of Al because some of the ligands of Si in the reaction system are replaced by Al, and Na is needed as a supplement to maintain a balanced charge. The distribution of Ca is relatively uniform, but its electrical pulse signal is weak and its light spots are dim, indicating that the small amount of cement mixed in the system is endogenous to small concentrations of hydrated calcium silicate and calcium silicate aluminate.

**Fig 16:**
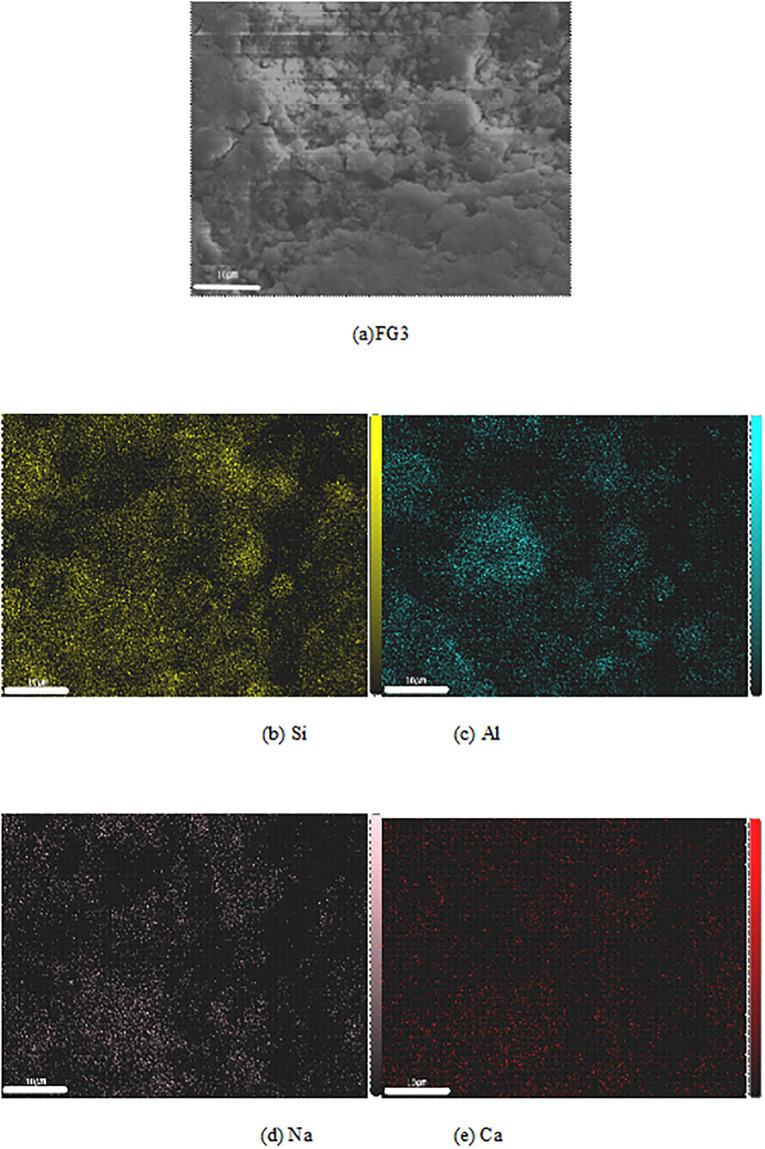
Elemental distribution of FG3.

The scanning results of FG3 are shown in Fig 16. Compared with FG0, the electrical pulse signal of Si is stronger at the overlapping position of Al and Si in FG3, and the proportion of Si at this position is larger. The binding mode between Al and FG0 is different, and the coordination number of Si replaced by Al is different. Na overlaps with Al in most positions, but its spots are dim. The distribution of Ca is relatively uniform, but due to its weak electrical pulse signal, it is difficult to find bright spots in the figure. Therefore, Ca is not dominant in the reaction system, but a large number of active Si and Al generate additional geopolymer gel, resulting in the macroscopic performance of these fly ash based geopolymers showing good compressive strength.

### 3.5. Pore structure

The change of the pore structure in the geopolymer system is an important factor as it affects the physical and mechanical properties of the geopolymer. Accordingly, the degree of mechanical activation of the fly ash particles and the number and size of pores inside the fly ash based geopolymer structure will change. Accordingly, FG0 and FG3 were compared. The internal pore characteristics of the geopolymer system after mechanical activation were measured, and the influence of grinding for 60 min on the microscopic pore structure of the fly ash based geopolymer was analyzed.

**Fig 17:**
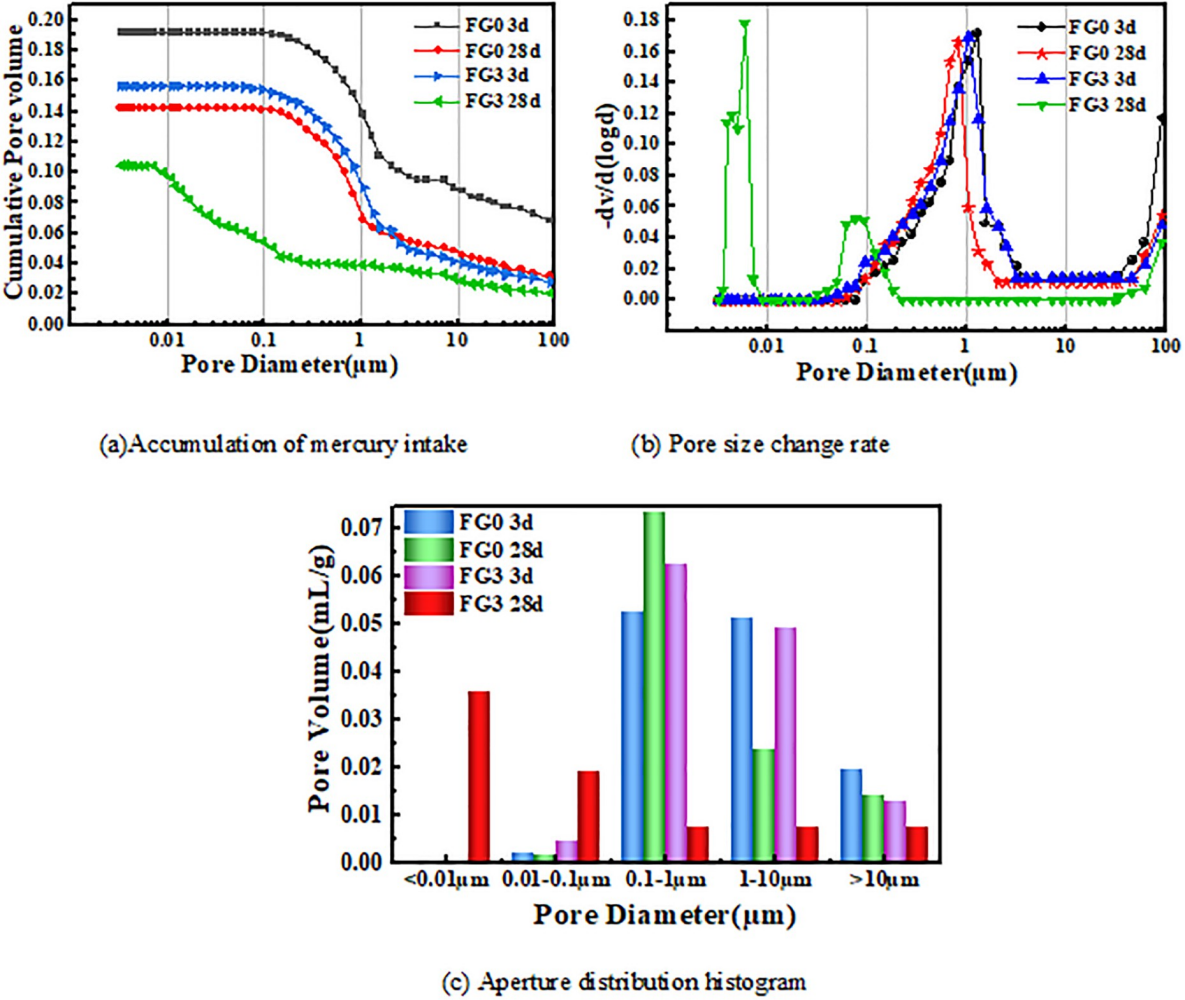
Aperture distribution maps.

The mercury intrusion experimental data of samples FG0 and FG3 is presented in Fig 17 and Table 4. The average pore size of FG0 decreased by approximately 37.5% and the porosity decreased by 16.6% as the curing duration increased from 3 d to 28 d, while the average pore size of FG3 decreased by 97.78%, and the porosity decreased by 33.2%. The pore size distribution map revealed that the widest pore size distribution area of FG0 was at 3d and the pore size increased from 0.1 to 10 μm. Although the pore size distribution shifted to the direction of the small pore size at 28 d, the change in range was not Significant. The pore size with internal pore size less than 0.1 accounted only for 0.74%, which was almost negligible. The pore size distribution of FG3 at 3 d was mainly concentrated in the 0.1–10 μm range, which is similar to that of FG0 at 3d, and the pore size slightly decreased. An internal pore size less than 0.01 μm accounted for 46.65% at 28 d, while a pore size less than 0.1 μm accounted for 71.6%. The area of the pore size distribution became narrower and shifted significantly to the direction of the smaller pore size. The proportion of the small pore significantly increased and the most probable pore size decreased. The grinding fly ash reduced the average pore size, porosity and most probable pore size of the fly ash based geopolymer. In particular, the effect of the grinding significantly improved the fly ash based geopolymer pore structure as the curing age increased as the dense glass phase shell on the surface of the fly ash particles was broken after grinding, which allowed the fly ash to react sufficiently in an alkali-activated environment. Furthermore, the fly ash was broken into a large number of fine particles, which filled the pores between the larger particles in the process of fly ash based geopolymer molding thus forming a micro-aggregate effect; this changed the pore structure distribution of the fly ash based geopolymer. The porosity of the system was reduced and the fly ash based geopolymer structure was denser, which is in accordance with the changes in the microstructure observed by SEM. The macroscopic performance of the material revealed that the compressive strength was considerably enhanced.

**Table 4:** Summary of MIP test results.

Sample	Total Intrusion Volume/mL∙g-1	Median Pore Diameter Volume/nm	Average Pore Diameter/nm	Porosity/%
FG0 3d	0.1921	3446.2	1204.7	27.1814%
FG0 28d	0.1422	1013.6	752.8	22.6602%
FG3 3d	0.1565	1230.1	685.1	24.5239%
FG3 28d	0.1044	105	15.2	16.3761%

### 3.6. Reaction degree

#### 3.6.1. Calculation of the reaction degree by the selective dissolution method

The quantity of unreacted fly ash can be obtained by measuring the residual mass of fly ash after alkali activation reaction by the selective dissolution method. The reaction degree of the fly ash based geopolymer was calculated according to Eq (2), which was prepared for further analysis to investigate the influence of the grinding degree of the fly ash on the reaction degree of fly ash based geopolymer.

$$Rex(\%)=(1-\frac{Ex}{\text{Fe}})*100\%$$ (2)

Where, Rex (%) represents the reaction degree of the fly ash based geopolymer, Fe represents the total mass of the fly ash based geopolymer sample before selective dissolution, and Ex represents the mass of the residue after selective dissolution.

**Fig 18:**
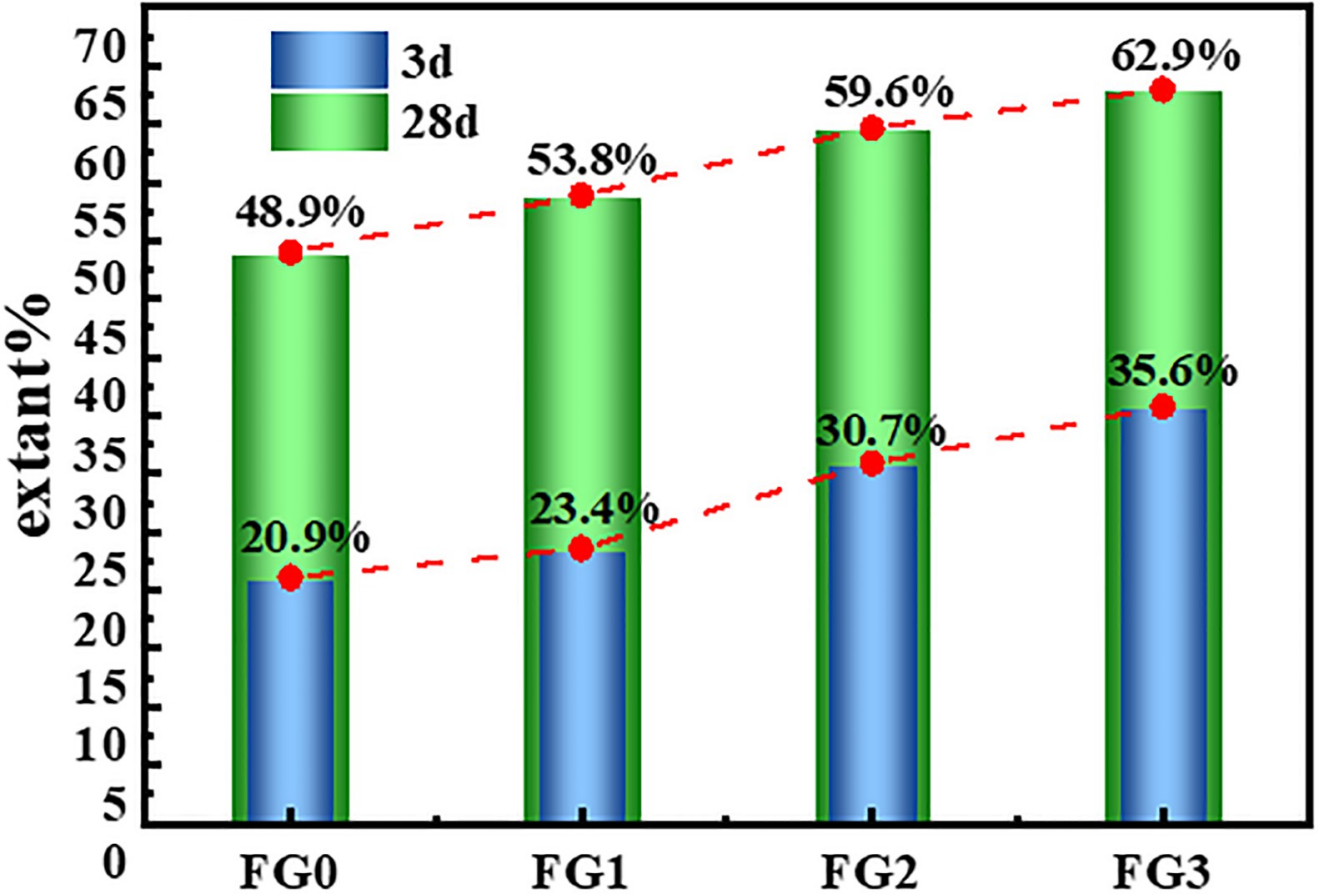
Effect of different grinding degrees of fly ash on the degree of geopolymer reaction.

The soluble silicate, partial soluble alkali, and alkaline salt in the fly ash based geopolymer were removed by a definite proportion of hydrochloric acid, and the residual amount of geopolymer prepared by fly ash with different grinding degrees after selective dissolution was determined. The influence of the grinding degree on the reaction degree of fly ash is shown in Fig 18. The results of Table 5 revealed that the amount of residue after selective dissolution of the prepared geopolymer decreased as the grinding degree of fly ash increased, and the reaction degree of fly ash based geopolymer increased as the grinding degree of the fly ash increased. However, the reaction degree of fly ash increased nonlinearly as the grinding degree increased (Fig 18). The growth rate of the reaction degree for fly ash increased upon grinding the fly ash for 40 min and slowed down upon grinding for 60 min. The broken fly ash particles steadily increased as the grinding time, which promoted the geological polymerization of the fly ash and improved the performance of the fly ash based geopolymer. However, the degree of grinding the fly ash particles into smaller particles gradually became limited upon grinding for long durations. Therefore, the increasing trend of the reaction degree for fly ash gradually slowed down with an increase for the grinding time, which is compatible with the increasing trend of the fly ash based geopolymer compressive strength.

**Table 5:** Quantification of the degree of geopolymer reaction in each group.

Sample	Residual mass at 3 d (g)	Si element content in residue at 3 d (mg/g)	Reaction degree) at 3 d (%)	Residual mass at 28 d (g)	Si element content in residue at 28 d (mg/g)	Reaction degree at 28 d (%)
FG0	7.91	177.62	20.36%	5.11	117.83	65.87%
FG1	7.66	118.76	48.43%	4.62	76.10	80.07%
FG2	6.93	121.47	59.8%	4.04	59.95	86.27%
FG3	6.44	92.04	66.40%	3.71	52.46	88.97%

#### 3.6.2. Mechanism analysis of increasing the reaction degree

The reaction process of geopolymers involves a complex chemical reaction process of alkali aluminosilicate polymers. Glukhovsky [43] was the first to propose in the middle of the 20^th^ century a linear model based on the alkali-excited reaction of low calcium aluminosilicates. The whole reaction process is divided into 3 stages. In the first stage, the fly ash particles are dissolved in a strong alkali environment, and the active [SiO_4_]^4-^ and [AlO_4_]^4-^ are dissolved out. In the second stage, the polycondensation reaction of [SiO_4_]^4-^ and [AlO_4_]^4-^ occurs, and the system begins to generate a gel-like reaction product. Finally, due to the further recombination and polymerization of the gel-like reaction product, semi-crystalline zeolite is formed which then begins to harden. As the curing time increases, Si and Al elements in the system continue to react in strong alkali environment to produce gel-like reaction products. As the gelatinous reaction product increases, the architecture becomes compact thus leading to an increase for the compressive strength.

**Fig 19:**
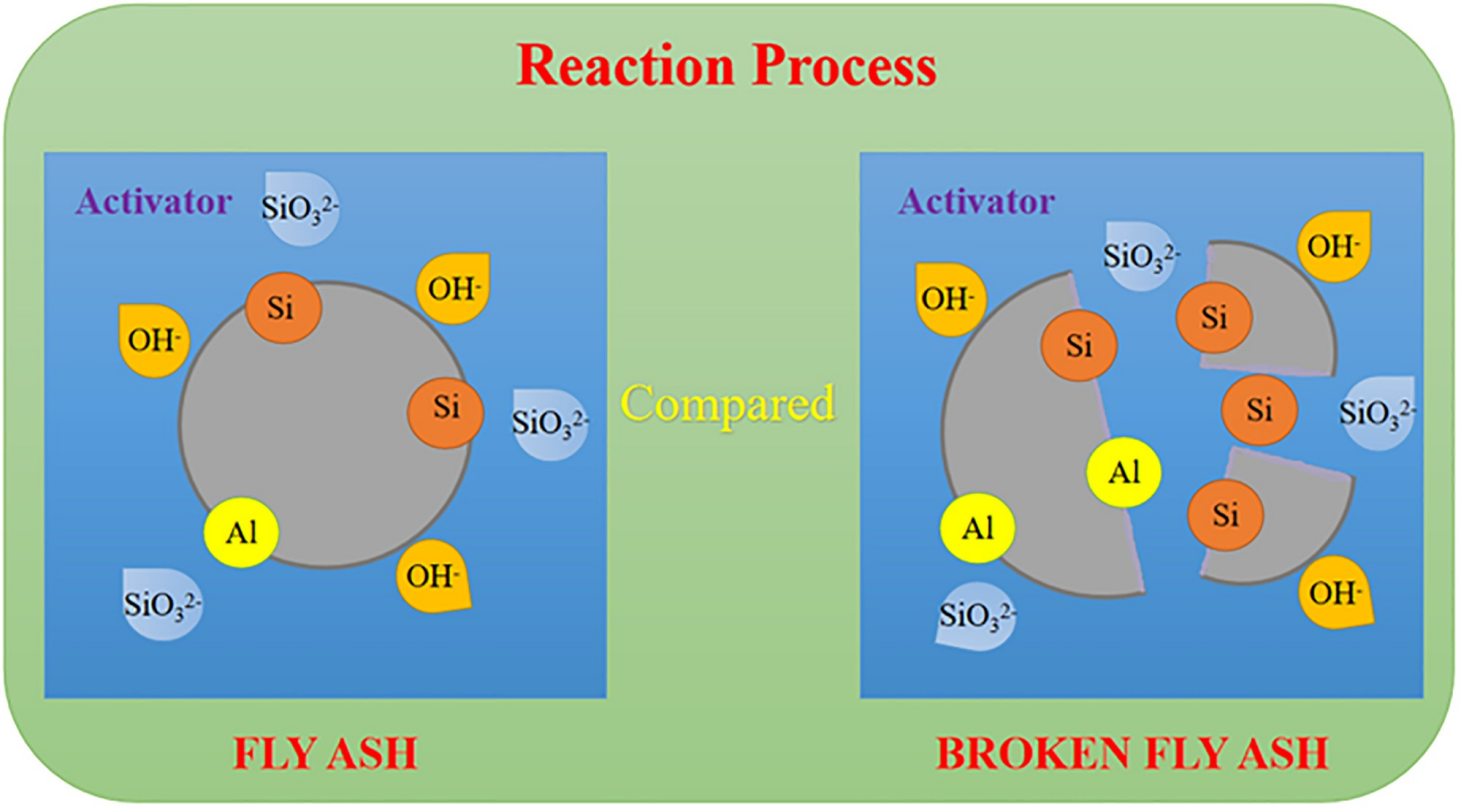
Reaction process comparison chart.

In this study, the effect of grinding on the fly ash is presented in Fig 19. After grinding, the dense glass phase shell on the surface of the fly ash particles that hinders the fly ash reaction was destroyed. The fly ash was broken into a large number of small particle size particles, which increased the specific surface area of fly ash after grinding. The contact surface of ground fly ash particles and alkali solution was larger than that of unground fly ash particles and the average particle size of fly ash particles after grinding decreased significantly. The fly ash particles with a small particle size dominated the whole system during the reaction with alkali solution. These factors accelerated the dissolution of active [SiO_4_]^4-^ and [AlO_4_]^4-^ in fly ash and promoted the depolymerization-polycondensation reaction of the geopolymer, which accelerated the reaction process of the whole system. The small fly ash particles formed after grinding maintained the dominant advantage and generated more reaction products during the curing process. This explains why the reaction degree of fly ash particles after grinding in strong alkali environment was greater than that of original fly ash particles. The compressive strength increased as the reaction degree increased and the internal structure of the system became compact, which is also highly consistent with the previous macroscopic and microscopic test results.

## 4. Conclusions

1. The average particle size tended to decrease with an increase of the grinding degree. The small particle size distribution increased, while the large particle size distribution tended to decrease. The average particle size of the fly ash decreased from 9.2448 μm to 5.8662 μm with a milling time in the range 0 to 60 min, corresponding to a decrease of approximately 36.5%.
2. The grinding degree of fly ash particles was positively correlated with the compressive strength of the geopolymer. At 28 d, the compressive strength of the geopolymer manufactured from fly ash after grinding for 60 min was approximately 109.7% higher than that of the geopolymer prepared by unground fly ash.
3. The small particles filled the pores between the large particles with an increase in the grinding degree, where the proportion of harmless pores significantly increased. The increase in the reaction products rendered the structure of the geopolymer compact. The average pore size was reduced by 737.6nm and porosity by 38.3% from 0min to 60min at 28d.
4. The dense glass phase shell on the surface of fly ash particles that hindered the reaction of fly ash was destroyed after grinding. Meanwhile, fly ash was broken into a large number of small particles. Increasing the specific surface area increased the contact area between the alkali solution and fly ash, which accelerated the reaction process and enhanced the reaction degree. The reaction degree of the geopolymer prepared by ground fly ash for 60 min was higher by 70.3% than that of the geopolymer prepared by the original fly ash at 3 d and was 28.6% higher at 28 d. However, the trend of increasing the reaction degree of fly ash was nonlinear with the increase of the grinding degree.

## Supporting information

S1 Fig XRD patterns of the raw materials. (XLSX)

S2 Fig Particle size distribution diagram. (XLSX)

S3 Fig Relationship between grinding time and average particle size of fly ash. (XLSX)

S4 Fig Net slurry fluidity. (XLSX)

S5 Fig Compressive strength of cement slurry and geopolymers. (XLSX)

S6 Fig XRD of geopolymer at 28. (XLSX)

S7 Fig Aperture distribution map. (XLSX)

S8 Fig Effect of different grinding degrees of fly ash on the degree of geopolymer reaction. (XLSX)
